# Lymphoepithelial cyst with sebaceous glands of the pancreas: a case report

**DOI:** 10.1186/s40792-016-0228-4

**Published:** 2016-09-15

**Authors:** Hiromitsu Maehira, Hisanori Shiomi, Koichiro Murakami, Hiroya Akabori, Shigeyuki Naka, Mitsuaki Ishida, Masaji Tani

**Affiliations:** 1 Department of Surgery, Shiga University of Medical Science, Seta-tsukinowacho, Otsu, Shiga 520-2192 Japan; 2 Department of Clinical Sciences and Laboratory Medicine, Kansai Medical University, Osaka, Japan

**Keywords:** Lymphoepithelial cyst, Sebaceous gland, Histogenesis

## Abstract

**Background:**

Lymphoepithelial cyst (LEC) of the pancreas is a rare benign tumor. LEC with sebaceous glands of the pancreas is extremely rare, and its histogenesis remains unclear.

**Case presentation:**

We present a 66-year-old man with an incidental finding of a cystic lesion at the neck of the pancreas. Pancreatic juice cytology results and elevated serum carbohydrate antigen 19-9 and Dupan-2 levels indicated that the cyst was a potential adenocarcinoma. Therefore, a pancreaticoduodenectomy was performed. Macroscopically, the tumor was a unilocular cyst with a thin transparent wall, filled with soft yellow material. Pathological findings showed that the cyst was lined with squamous epithelium, accompanied by dense lymphoid tissue with scattered germinal centers. There were no hair follicles, but sebaceous glands were present in the lymphoid tissue just beneath the squamous epithelium. Therefore, the histopathological diagnosis was an LEC with sebaceous glands of the pancreas. Furthermore, the squamous epithelium surrounding the cyst was pathologically continuous with the tubular structure, indicating that the tubular structure transitioned into the squamous epithelium.

**Conclusions:**

We report an extremely rare case of LEC with sebaceous glands of the pancreas. Moreover, the pathological findings, which showed that the tubular structure transitioned into the squamous epithelium, suggested that this was squamous metaplasia. In order to investigate the histogenesis of LEC of the pancreas, the pathological findings must be evaluated.

## Background

A lymphoepithelial cyst (LEC) is a lesion that most commonly appears on the lateral neck and the parotid gland. LEC of the pancreas is a stratified squamous-lined cyst filled with keratinous material and surrounded by dense lymphoid tissue. LEC of the pancreas is a rare lesion and its histogenesis remains unclear. In particular, LEC with sebaceous glands is extremely rare [1]; sebaceous glands in a non-skin organ are often found in ectodermal organs, but they are rarely found in endodermal organs such as the pancreas. We herein present a case of LEC with sebaceous glands of the pancreas, in which the tubular structure/squamous epithelium change was seen on the pathological examination.

## Case presentation

A 66-year-old man was referred to our hospital for further evaluation of a cystic lesion in the pancreatic head that was detected on computed tomography (CT) of the abdomen. He had liver dysfunction due to hepatitis C infection and diabetes mellitus. He had no history of abdominal surgery, trauma, or pancreatitis. Laboratory findings showed that serum levels of amylase and lipase were within normal limits. Carcinoembryonic antigen and s-pancreatic-1 antigen levels were within normal limits, but carbohydrate antigen 19-9 (CA19-9) and Dupan-2 levels were elevated (CA19-9, 55 U/ml (normal level, <37 U/ml); Dupan-2, 410 U/ml [normal level, <150 U/ml]).

**Fig. 1:**
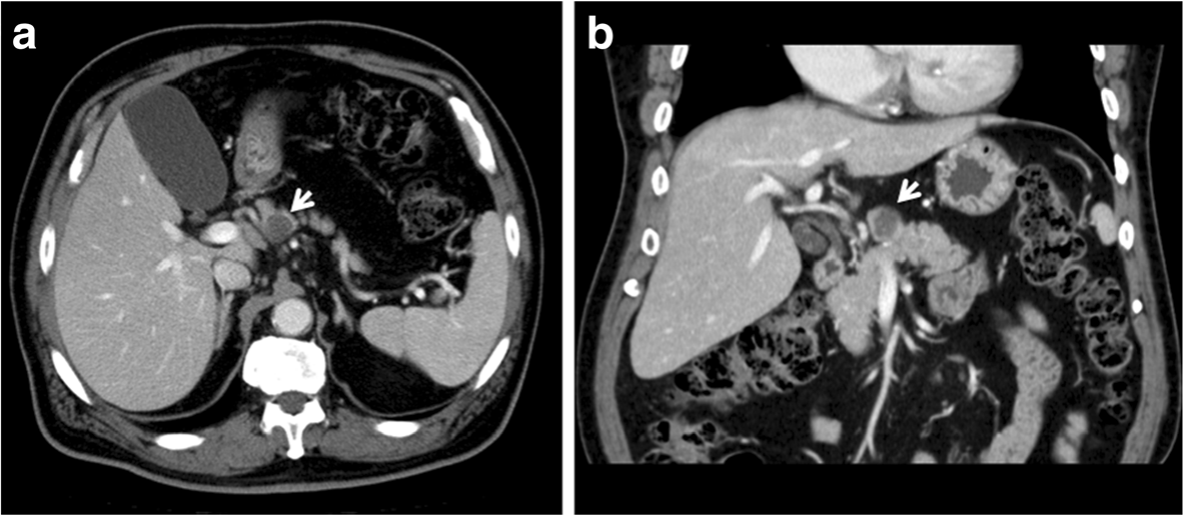
Abdominal contrast-enhanced computed tomography. Abdominal contrast-enhanced computed tomography showing a clear, round, low-density mass, 20 mm in size, protruding from the neck of the pancreas (*arrows*). **a** Axial view and **b** coronal view

**Fig. 2:**
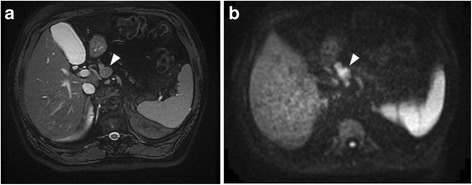
Magnetic resonance imaging of the abdomen. **a** T2-weighted image showing a clear, round, mass in which the intensity was lower than the intensity of water. **b** Diffusion-weighted image showing a high-intensity mass (*arrowheads*)

**Fig. 3:**
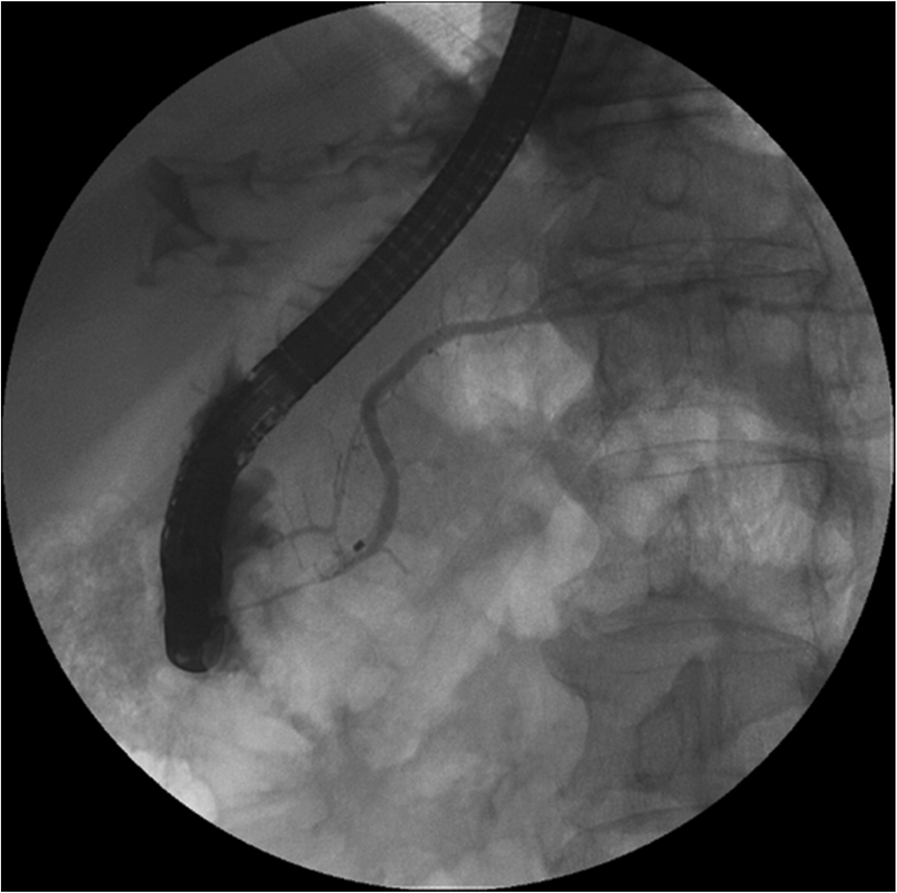
Endoscopic retrograde cholangiopancreatography. Endoscopic retrograde cholangiopancreatography showing no abnormality in the main pancreatic duct

**Fig. 4:**
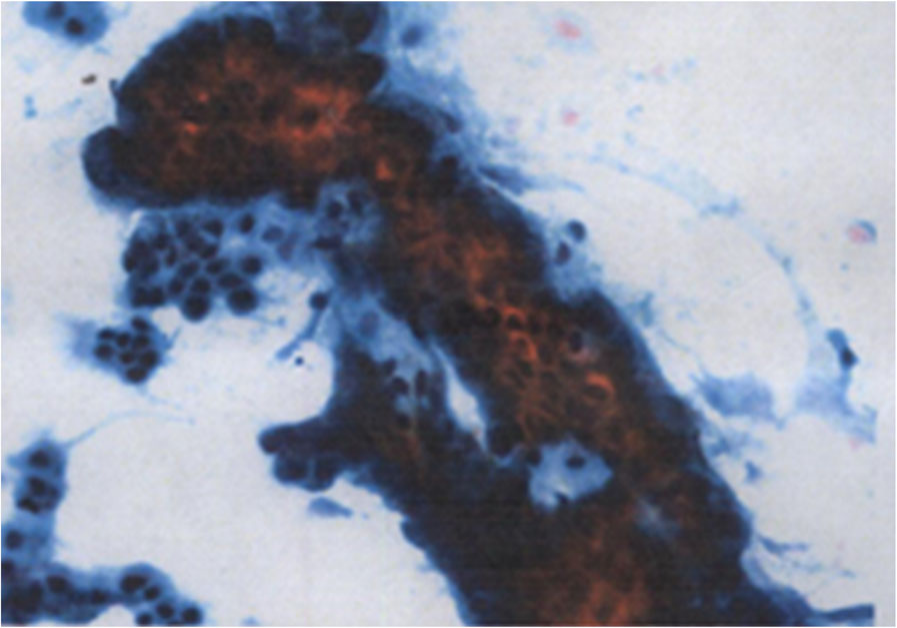
Cytology of pancreatic juice. Cytology of pancreatic juice showing large cell clusters with small nuclei and strong proliferation, indicating a suspected adenocarcinoma

Abdominal contrast-enhanced CT showed a low-density mass, 20 mm in size, at the neck of the pancreas. The mass was clear and round, with uniform content and no septa. The mass appeared to be protruding from the neck of the pancreas (Fig. 1). Magnetic resonance imaging (MRI) showed a round mass in which the intensity was lower than the intensity of water on T2-weighted images. The tumor showed high intensity on diffusion-weighted images (Fig. 2). Endoscopic retrograde cholangiopancreatography showed no abnormality in the main pancreatic duct, and there was no evident continuity between the tumor and the main pancreatic duct (Fig. 3). The pancreatic juice contained high levels of mucin, and cytological examination showed large cell clusters with no nuclear atypia. These clusters showed high levels of proliferation, although the nuclei were relatively small (Fig. 4). The tumor was diagnosed as a suspected adenocarcinoma with a retention cyst, and a pancreaticoduodenectomy was performed.

**Fig. 5:**
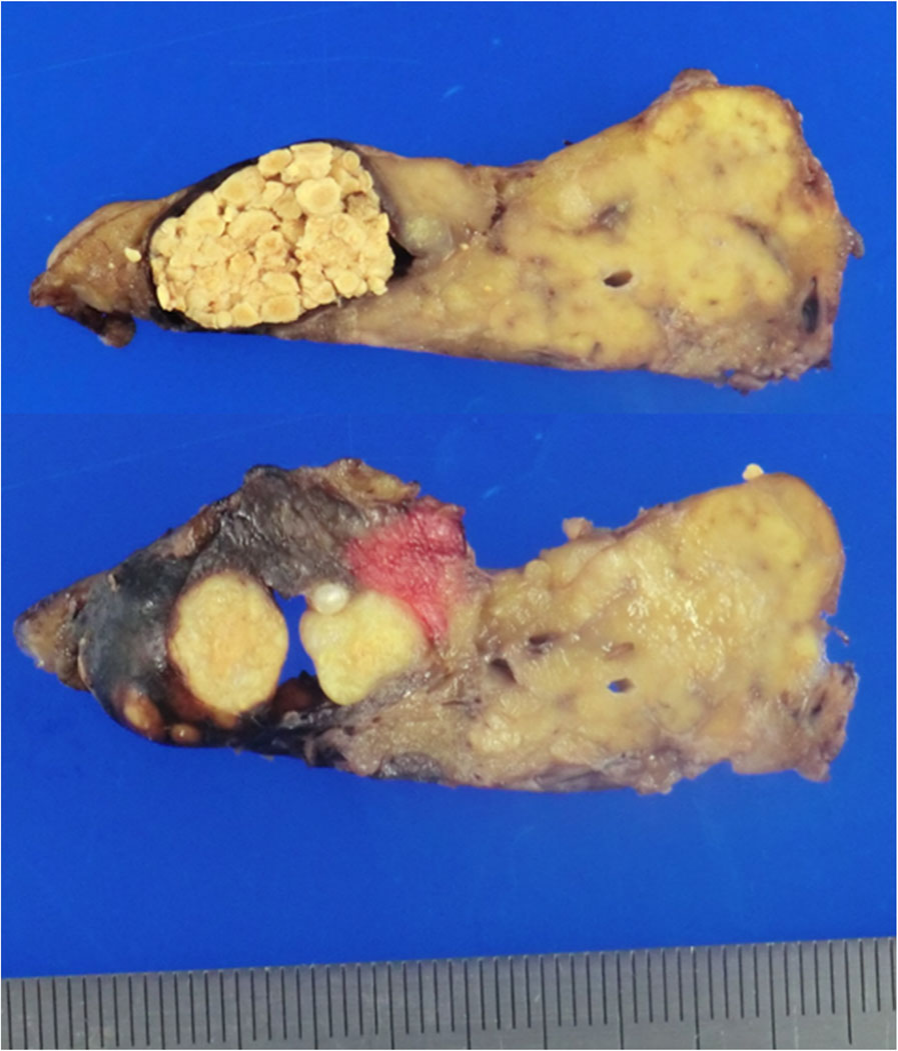
Cross sections of the resected specimen. The tumor size was 20 mm in diameter and demonstrated a unilocular cyst with a thin transparent wall, filled with soft yellow material

**Fig. 6:**
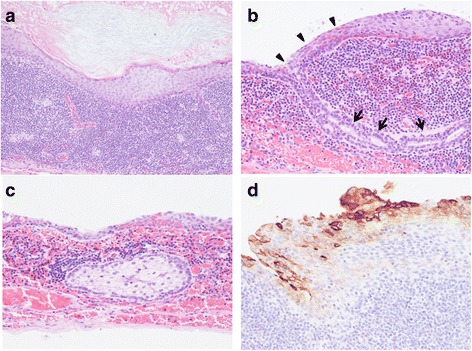
Pathological findings. **a** Cyst surrounded by squamous-lined epithelium, accompanied by dense lymphoid tissue with scattered germinal centers. Hematoxylin-eosin staining, original magnification ×40. **b** Tubular structure (*arrows*) continuous with the squamous epithelium (*arrowheads*). Hematoxylin-eosin staining, original magnification ×400. **c** Sebaceous glands present in the lymphoid tissue just beneath the squamous epithelium. Hematoxylin-eosin staining, original magnification ×200. **d** Immunohistochemistry showing for CA19-9-positive cells in the squamous epithelium

Macroscopically, the tumor was 20 mm in size. It consisted of a unilocular cyst with a thin transparent wall, filled with soft yellow material (Fig. 5). Pathological findings showed that the cyst was lined with squamous epithelium, accompanied by dense lymphoid tissue with scattered germinal centers (Fig. 6a). The squamous epithelium surrounding the cyst was continuous with the tubular structure, potentially representing the transition of the tubular structure into squamous epithelium (Fig. 6b). There were no hair follicles, but sebaceous glands were present in the lymphoid tissue just beneath the squamous epithelium (Fig. 6c). There was no evidence of dysplasia or malignancy. Immunostaining showed that the squamous epithelium expressed CA19-9 (Fig. 6d). The histopathological diagnosis was a benign lymphoepithelial cyst with sebaceous glands of the pancreas.

Although delayed gastric emptying occurred postoperatively, the patient was discharged on postoperative day 28. Eighteen months after surgery, there were no signs of recurrence or other lesions such as pancreatic cancer or intraductal papillary mucinous neoplasm on abdominal contrast-enhanced CT.

### Discussion

We resected an LEC with sebaceous glands of the pancreas, which is extremely rare. In addition, the pathological findings revealed the histogenesis of the LEC. These findings showed squamous metaplasia that subsequently protruded from the pancreatic parenchyma into a peripancreatic lymph node.

The first description of a squamous-lined cyst of the pancreas, which was filled with keratinous material and surrounded by dense lymphoid tissue, was reported by Luchirath et al. in 1985 [2]. In 1987, Truong et al*.* proposed the term “LEC of the pancreas” [3]. Diane et al*.* reviewed 117 cases of LEC of the pancreas between 1987 and 2012: 78 % of the patients were men; serum CA19-9 levels were elevated in 50 % of cases; and LEC of the pancreas could be correctly diagnosed on fine-needle aspiration (FNA) biopsy in 21 % of cases [4]. LEC of the pancreas shows variable imaging findings because it has both liquid and solid components. MRI is useful for the diagnosis of cystic tumors; however, cysts with viscous fluid show low intensity on T2-weighted images and high intensity on diffusion-weighted images. It is very difficult to diagnose LEC correctly or to rule out malignancy on the basis of the preoperative imaging findings. Resection is unnecessary for benign non-neoplastic disease, including LEC. FNA can be used to make a preoperative pathological diagnosis; however, if the cyst is a malignant tumor, there is a risk of peritoneal seeding with FNA. Careful examination and informed consent are necessary before FNA of a cystic tumor.

In this case, the cyst was lined with stratified squamous epithelium with dense lymphoid tissue underneath. Furthermore, a sebaceous gland was embedded in the lymphoid tissue just beneath the squamous epithelium. These results suggested a potential dermoid cyst. However, we diagnosed the cyst as an LEC of the pancreas with sebaceous differentiation owing to the lack of hair follicles, fewer sebaceous glands, and the presence of dense lymphoid tissue. Only seven cases of LEC of the pancreas with sebaceous differentiation have been reported to date [1, 5–10].

The clinical features of the previously reported patients are summarized in Table 1. All these patients were middle-aged men. The median cyst size was 47.5 mm (range, 20–100 mm). The location and the loculation of the cysts varied. FNA was performed in four cases, but only one case was correctly diagnosed preoperatively. These results highlight the difficulties associated with preoperative diagnosis of LEC of the pancreas. Slightly elevated serum CA19-9 levels were present in four cases; CA19-9 levels were not determined in the other cases. This highlights another difficulty in the diagnosis, because elevated serum CA19-9 levels may lead to a false diagnosis of a malignant tumor.

**Table 1:** Clinical features of previously reported lymphoepithelial cysts with sebaceous glands of the pancreas

	Author	Age (years)/sex	Symptom	Pancreatic location	Max. Size	Loculation	FNA	Serum CA19-9 (U/ml)	Preoperative diagnosis
1	Fitko [5]	60/male	Abdominal pain	Body	45 mm	Uni	Yes	N/A	A diagnosis could not be made
2	Koga [6]	62/male	None	Head	52 mm	Multi	No	N/A	Cystadenocarcinoma
3	Rino [7]	58/male	None	Head	50 mm	Multi	Yes	39	Epidermoid cyst
4	Fukukura [8]	70/male	Diarrhea	Tail	100 mm	Uni	No	N/A	Not described
5	Fujiwara [9]	60/male	None	Tail	40 mm	Uni	No	98	Mucinous cystic neoplasm
6	Hebert [10]	48/male	None	Body	50 mm	Uni	Yes	N/A	LEC with sebaceous differentiation
7	Nakamura [1]	67/male	None	Body	42 mm	Multi	Yes	69.2	Cystic neoplasm
8	Our case	66/male	None	Head	20 mm	Uni	No	55	CIS or IPMC

*Abbreviations*: *FNA* fine needle aspiration, *CA19-9* serum carbohydrate antigen 19-9, *N/A* information not available, *LEC* lymphoepithelial cyst, *CIS* carcinoma in situ, *IPMC* intraductal papillary mucinous carcinoma

Sebaceous glands in a non-skin organ, i.e., ectopic sebaceous glands, are often found in ectodermal organs. However, ectopic sebaceous glands or neoplastic lesions with sebaceous glands rarely occur in the endodermal organs, such as the thyroglossal duct, larynx, esophagus, or thymus [11]. The pancreas is also an endodermal organ. Sebaceous gland differentiation at various sites may be due to the multipotentiality of the germinative cells of the squamous epithelium [5]; however, few details are available owing to the low number of reported cases.

The histogenesis of LEC of the pancreas remains unclear. Three theories have been proposed: (1) the lesion arises from a misplaced portion of the branchial cleft that fuses with pancreatic anlage during embryogenesis, (2) the lesion represents squamous metaplasia of an obstructed and dilated pancreatic duct that subsequently protrudes from the pancreatic parenchyma into a peripancreatic lymph node, and (3) the lesion arises from a benign epithelial inclusion or ectopic pancreas in a peripancreatic lymph node and subsequently encroaches into the pancreatic tissue [3]. The hypothesis that the lesion arises from a misplaced portion of the branchial cleft may suggest distinctive histologic features consistent with an anatomic relationship with the pancreas. However, this has not been confirmed by any embryologic evidence. Another hypothesis that the lesion arises from a benign epithelial inclusion or ectopic pancreas is likely to have arisen due to its similarity to the more common salivary lymphoepithelial cyst [12]. However, the ectopic pancreas in peripancreatic lymph nodes is extremely rare [13], and this hypothesis cannot account for the observed continuity with the pancreatic parenchyma. In the present case, the cyst was continuous with the pancreatic parenchyma, histopathologically. Furthermore, it was indicated that the cyst wall represented squamous metaplasia because the transition of the tubular structure into squamous epithelium was seen pathologically. Furthermore, CA19-9-positive cells were observed in the squamous epithelium on immunostaining. Therefore, it is likely that the histogenesis in this case may be considered squamous metaplasia of the glandular epithelium of the pancreas, which subsequently protruded from the pancreatic parenchyma into the peripancreatic lymphoid tissue.

## Conclusions

Here, we report an extremely rare case of LEC with sebaceous glands of the pancreas. We believe that this is the first report to demonstrate the histogenesis of LEC of the pancreas in the pathological findings. To further investigate the histogenesis of LEC of the pancreas, it will be necessary to evaluate pathological findings in detail by accumulating additional cases.

## Abbreviations

CA19-9: Carbohydrate antigen 19-9
CT: Computed tomography
FNA: Fine-needle aspiration biopsy
LEC: Lymphoepithelial cyst
MRI: Magnetic resonance imaging.
